# Genetic diversity and population structure of a Peruvian cattle herd using SNP data

**DOI:** 10.3389/fgene.2023.1073843

**Published:** 2023-03-10

**Authors:** Flor-Anita Corredor, Deyanira Figueroa, Richard Estrada, Wilian Salazar, Carlos Quilcate, Héctor V. Vásquez, Jhony Gonzales, Jorge L. Maicelo, Percy Medina, Carlos I. Arbizu

**Affiliations:** ^1^ Dirección de Desarrollo Tecnológico Agrario, Instituto Nacional de Innovación Agraria (INIA), Lima, Peru; ^2^ Facultad de Ingenierŕa Zootecnista, Agronegocios y Biotecnología, Universidad Nacional Toribio Rodríguez de Mendoza de Amazonas, Amazonas, Peru; ^3^ Laboratorio de Biología Molecular, Universidad Nacional de Frontera, Piura, Peru

**Keywords:** cattle breeds, genotypes, diversity, genomics, NGS

## Abstract

New-generation sequencing technologies, among them SNP chips for massive genotyping, are useful for the effective management of genetic resources. To date, molecular studies in Peruvian cattle are still scarce. For the first time, the genetic diversity and population structure of a reproductive nucleus cattle herd of four commercial breeds from a Peruvian institution were determined. This nucleus comprises Brahman (*N* = 9), Braunvieh (*N* = 9), Gyr (*N* = 5), and Simmental (*N* = 15) breeds. Additionally, samples from a locally adapted creole cattle, the Arequipa Fighting Bull (AFB, *N* = 9), were incorporated. Female individuals were genotyped with the GGPBovine100K and males with the BovineHD. Quality control, and the proportion of polymorphic SNPs, minor allele frequency, expected heterozygosity, observed heterozygosity, and inbreeding coefficient were estimated for the five breeds. Admixture, principal component analysis (PCA), and discriminant analysis of principal components (DAPC) were performed. Also, a dendrogram was constructed using the Neighbor-Joining clustering algorithm. The genetic diversity indices in all breeds showed a high proportion of polymorphic SNPs, varying from 51.42% in Gyr to 97.58% in AFB. Also, AFB showed the highest expected heterozygosity estimate (0.41 ± 0.01), while Brahman the lowest (0.33 ± 0.01). Besides, Braunvieh possessed the highest observed heterozygosity (0.43 ± 0.01), while Brahman the lowest (0.37 ± 0.02), indicating that Brahman was less diverse. According to the molecular variance analysis, 75.71% of the variance occurs within individuals, whereas 24.29% occurs among populations. The pairwise genetic differentiation estimates (F_ST_) between breeds showed values that ranged from 0.08 (Braunvieh vs. AFB) to 0.37 (Brahman vs. Braunvieh). Similarly, pairwise Reynold’s distance ranged from 0.09 (Braunvieh vs. AFB) to 0.46 (Brahman vs. Braunvieh). The dendrogram, similar to the PCA, identified two groups, showing a clear separation between *Bos indicus* (Brahman and Gyr) and *B. taurus* breeds (Braunvieh, Simmental, and AFB). Simmental and Braunvieh grouped closely with the AFB cattle. Similar results were obtained for the population structure analysis with *K* = 2. The results from this study would contribute to the appropriate management, avoiding loss of genetic variability in these breeds and for future improvements in this nucleus. Additional work is needed to speed up the breeding process in the Peruvian cattle system.

## 1 Introduction

Livestock production around the world is a large sector with an important contribution of 40% and 20% to agriculture production in developed and developing countries, respectively (Herrero et al., 2013; Baltenweck et al., 2020; FAO, 2022). A large part of Peru’s livestock economy revolves around cattle production (León-Velarde and Quiroz, 2004). According to the latest Peruvian National Agricultural Census (Instituto Nacional de Estadística e Informática, 2012), Peruvian creole cattle (PCC) is the most predominant cattle population (64.03%). PCC is prevalent in the Andean sector of the country, where it has been adapted to the highlands climate conditions (Quispe, 2016; Delgado et al., 2019). However, in comparison with exotic breeds, PCC achieves smaller body weights and milk production records (Espinoza and Urviola, 2005; Dipas Vargas, 2015; Ruiz et al., 2021).

Due to the low productivity, small farmers breeding strategy is to crossbred PCC with other specialized breeds in order to take advantage of the heterosis effect (Seré et al., 1996; W. et al., 2019). Nowadays, there are a high availability of bovine breeds that can be used to improve milk, meat or double purpose production (Thibier and Wagner, 2002; Mebratu et al., 2020). However, Peruvian initiatives are lacking the understanding of the genetics behind. Genetic diversity knowledge is essential for the effective management of genetic resources (Groeneveld et al., 2010; Hoban et al., 2013). In recent years the availability of genotyping technology has become affordable in livestock allowing to increase genetic studies (Mukhopadhyay et al., 2020). As a result, SNP markers are becoming increasingly common for diversity analysis and population structure studies (Morin et al., 2009; Haasl and Payseur, 2011). SNP markers have the advantage of being abundant in the genome, as well as the ability to be automated through high-through genotyping panels (Beuzen et al., 2000; Vignal et al., 2002).

In developing countries, nucleus breeding systems represent a good strategy for animal genetic improvement for ruminants. Concentrating nucleus cattle in one or a few herds to disseminate genetic material to other populations is helpful (Kiwuwa, 1992; Schrooten and van Arendonk, 1992). In 1993, a Peruvian government herd composed of Brahman, Braunvieh, Gyr and Simmental breeds was established with the aim to develop reproductive technology research, such as artificial insemination and embryo transfer. Currently, the herd is distributing semen straws and embryos to producers’ associations in order to disseminated specialized cattle breed genetics. This herd is been called a genetic nucleus herd, however, there is scarcity of data available in pedigrees and production records. Therefore, this study aims to provide understanding of the genetic diversity among the breeds on this herd, and its population structure, including a PCC group on the study. We expect to genomic characterize the nucleus using SNP markers, by obtaining genetic diversity and population structure parameters.

## 2 Materials and methods

### 2.1 Animal sampling and DNA extraction

A total of 63 blood samples were collected from four commercial breeds of taurus (Braunvieh and Simmental) and indicus cattle (Brahman and Gyr). According to their pedigree, up to grandfathers, genetic origins for Brahman and Gyr were predominantly from Brazil; for Braunvieh, Switzerland and Colombia; while for Simmental was Germany (Supplementary Table S1). Blood sampling was performed from a government herd, the Donoso Agricultural Research Station (EEA Donoso in Spanish) located in Huaral, Lima (128 masl; 11°31′18″ S and 77°14′06″ W). Pedigree was checked to avoid sampling from related individuals, animals were not siblings or had a parental relationship. Blood samples were collecterelated individuals, animals were not siblings or have a parentald from the epidural vein using a vacutainer containing EDTA as an anticoagulant and were immediately transferred to the laboratory for DNA extraction. Additionally, we got access to 12 hair samples that were collected from the tail of individuals that were considered as “Arequipa fighting bull” (AFB), which are bovines from Arequipa region (2,335 masl; 15°29′58″ S and 72°21′36″ W). Most of these individuals were selected as they possessed most of the morphological characteristics of a PCC as identified by their owners, where its body is unbalanced with the topline being higher on the front and becoming smaller toward the rear. For the PCC, the hooks to pin are lower-level hipped when compared to other breeds of cattle, dairy or beef. The length of the body is shorter, as is the topline. Colors of hair have multiple variations. The diversity of colors ranges from a total color cover to mixed ones and spotting ones.

We extracted genomic DNA from whole blood and hair samples with the Wizard Genomic DNA Purification Kit (Fitchburg, WI, United States) following the manufacturer’s instructions. The quality and quantity of genomic DNA were assessed using agarose gel electrophoresis and a Nanodrop spectrophotometer (Model ND 2000, Thermo Fisher Scientific, Wilmington, DE, United States) prior to genotyping. In addition, 40 genotypes from reference breeds were included in the analyses. The reference breeds were sourced from Decker et al. (2014) and the world reference dataset in Web-Interfaced Next-Generation Database (WIDDE) database (Sempéré et al., 2015). Breeds included were Brahman, Braunvieh, Gyr, and Simmental.

### 2.2 SNP genotyping and quality control

DNA samples were genotyped using Illumina Bovine HD Genotyping BeadChip and Illumina GGP Bovine 100K BeadChip with the help of the commercial genotyping service provider (Neogen, Geneseek, NL, United States). Female individuals were genotyped with the GGPBovine100K and males with the BovineHD. The Bovine HD and 100K chips possess 777,962 and 95,256 SNPs, respectively, uniformly spanning over the entire bovine genome. A total of 87,669 common markers between both SNP panels were used for the following analysis. From the total of 71 animals sampled, we discarded the ones with a genotype call rate minor to 85% (Purfield et al., 2016). A total of 18 samples were discarded before starting the SNP quality control. We started the SNP quality control with 53 animals and 47 remained for the following analysis after quality control.

SNPs quality control was performed using the PLINK v1.9 program (Purcell et al., 2007). SNPs assigned to sex chromosomes and those lacking genomic locations were excluded from the analysis. SNPs with missing genotypes in more than 10% of individuals, missing rate per SNP of 10%, and minor allele frequency (MAF) lower than 0.05 were excluded. However, SNP filtering based on the Hardy–Weinberg equilibrium was not performed since we expected Hardy–Weinberg deviations in the studied populations due to their small and possibly sub-structured population and genetic drift (Chen et al., 2017). We used 80,178 autosomal SNPs that remained after applying filtering criteria to assess genetic diversity. Additionally, linkage disequilibrium pruning, using the parameter indep (50 5 2), was performed before the population structure analysis. A total of 16,345 SNPs were obtained after pruning for LD.

### 2.3 Genetic diversity

To assess the genetic diversity within the studied population we used different genetic diversity parameters. The proportion of polymorphic SNPs (Pn), MAF, expected heterozygosity (He), observed heterozygosity (Ho), and inbreeding coefficient (F_IS_) were estimated using R package *dartR* (Gruber et al., 2018). The distribution of MAF was grouped into five different categories based on the frequency of rare alleles (0 < MAF ≤0.1), intermediate alleles (0.1 < MAF ≤0.2, 0.2 < MAF ≤0.3, and 0.3 < MAF ≤0.4), and common alleles (0.4 < MAF ≤0.5).

### 2.4 Population structure

Different approaches were employed to investigate the genetic structure among the cattle populations of the EEA Donoso herd, and assess their relationships with the AFB cattle. First, an analysis of molecular variance (AMOVA) was performed with ARLEQUIN v.3.5.2 software (Excoffier and Lischer, 2010), with the locus by locus option and 1,000 permutations. PGDSpider v.2.1.1.5 software (Lischer and Excoffier, 2012) was used to convert files between PLINK and Arlequin formats. We used ARLEQUIN to assess the divergence among breeds. Genetic differentiation among breed (F_ST_) fixation indices were calculated using 20,000 permutations and a significance level of 0.05. Also, Reynold’s distance was performed. Second, a principal component analysis (PCA), and a discriminant analysis of principal components (DAPC) were perform with PLINK. The *factorextra* (Kassambara and Mundt, 2017) and *adegenet* (Jombart and Collins, 2017) R packages were used to generate eigenvectors and eigenvalues, and the outputs were visualized using the package *ggplot2* (Gómez-Rubio, 2017). For PCA and DAPC, animals form the referenced population were included in the analyses. Third, an assessment of population genetic structure was performed using the default settings of ADMIXTURE v.1.3 software (Alexander et al., 2009). The most appropriate K value was selected after considering 10-fold cross-validations whereby the best K exhibits low cross validation error compared to other K values (Alexander and Lange, 2011). Finally, a Neighbor-Joining tree was constructed using *vcfR* (Knaus and Grünwald, 2017), *pegas* (Paradis, 2010), and *ape* (Paradis and Schliep, 2019) packages in R. Additionally, 1,000 bootstrap replicates were conducted.

## 3 Results

### 3.1 Genetic diversity analysis

The results of the genetic diversity parameters calculated for the different cattle breed groups genotyped are summarized in Table 1. Most of the breeds show a high Pn, varying from 51.42% in Gyr to 97.58% in AFB. The highest mean MAF value was observed in AFB (0.32 ± 0.13), and the lowest value was observed in Gyr (0.13 ± 0.16) with a mean value of 0.23 across populations. The He ranged from 0.33 (Brahman) to 0.41 (AFB). The highest observed heterozygosity was observed in Braunvieh (0.43 ± 0.01), while the lowest was in Brahman (0.37 ± 0.02). The Ho was greater than the He and the inbreeding coefficient was negative for the breeds, except for AFB.

**TABLE 1:** Genetic diversity, showing the name of the breed, sample size (N), the proportion of polymorphic SNPs (Pn), minor allele frequency (MAF), expected heterozygosity (He), observed heterozygosity (Ho), and inbreeding coefficient (F_IS_).

Population	N	Pn (%)	MAF	He	Ho	F_IS_
Brahman	9	62.18	0.15 ± 0.16	0.33 ± 0.01	0.37 ± 0.02	−0.07
Braunvieh	9	92.06	0.27 ± 0.15	0.38 ± 0.01	0.43 ± 0.01	−0.07
Simmental	15	95.37	0.28 ± 0.14	0.38 ± 0.01	0.41 ± 0.02	−0.05
Gyr	5	51.42	0.13 ± 0.16	0.35 ± 0.01	0.41 ± 0.02	−0.05
AFB	9	97.58	0.32 ± 0.13	0.41 ± 0.01	0.42 ± 0.04	0.03

**FIGURE 1:**
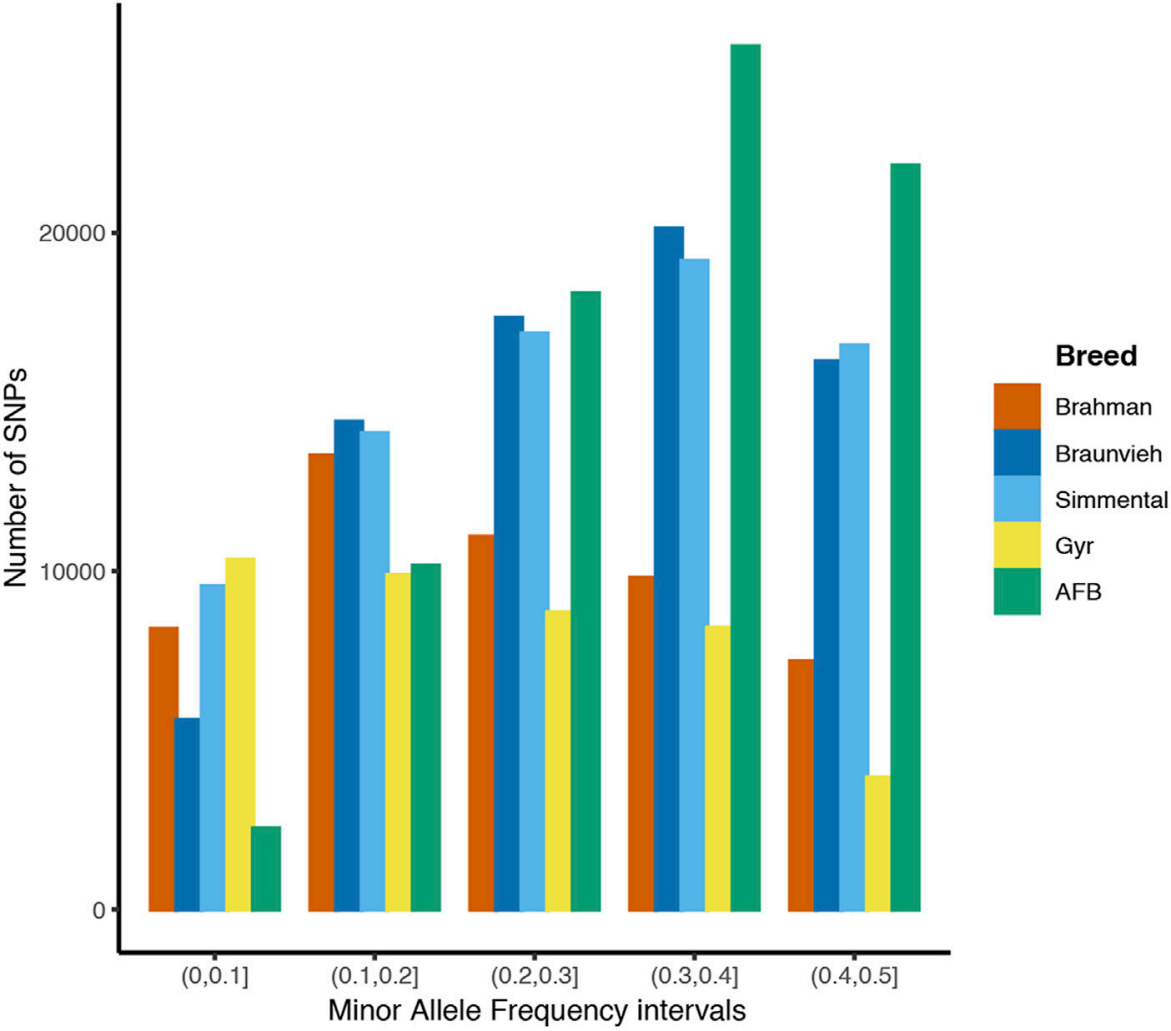
Distribution of minor allele frequency for each cattle breed.

Minor allele frequency distribution for different categories is shown in Figure 1. Among the five cattle breeds, AFB (25,504) and Gyr (3,903) showed the highest and the lowest count of SNPs when MAF greater than or equal to 0.3.

The Gyr breed had a higher count of SNPs in the lowest MAF interval (MAF≤0.1) compared to the counts of SNPs in the higher MAF intervals. The count of SNPs for the Braunvieh, AFB, and Simmental cattle breeds was shown to be higher as the MAF interval increased. For the Brahman and Gyr breeds the count of SNPs decreased.

### 3.2 Population structure

The AMOVA results (Table 2) showed that the most important part of the genetic variation (75.71%) was observed within the cattle breeds and variability among the cattle breeds was 24.29%. Also, pairwise F_ST_ and Reynold’s distance among all populations were estimated (Table 3). The pairwise F_ST_ estimates among breeds ranged from 0.08 (AFB-Braunviehpair) to 0.37 (Braunvieh-Brahman pair). The pairs Braunvieh-Brahman and Braunvieh-Gyr showed high pairwise F_ST_ values, with 0.37 and 0.36, respectively. Furthermore, the pairs Braunvieh-AFB, and AFB-Simmental showed the lowest pairwise F_ST_ values, with 0.08 and 0.09, respectively. The pairwise Reynold’s distance showed a pattern similar to the one obtained with the F_ST_ statistics, with values ranging from 0.09 (AFB-Braunvieh pair) to 0.46 (Braunvieh-Brahman pair). The pairs Brahman-Braunvieh and Braunvieh-Gyr showed high pairwise Reynold’s distance values, with 0.46 and 0.44, respectively. Furthermore, the pairs AFB-Braunvieh, and AFB-Simmental showed the lowest pairwise F_ST_ values, with 0.09, respectively each.

**TABLE 2:** Analysis of molecular variance among five cattle breeds.

Source of variation	Degree of freedom	Sums of squares	Variance component	% of variations
Among population	4	365574.76	4,301.64	24.29
Within individuals	89	1184932.66	13411.11	75.71
Total	93	1550507.41	17712.74	

**TABLE 3:** Estimates of the pairwise genetic differentiation statistic (F_ST_ statistics; below the diagonal) and the Reynold’s genetic distance (above the diagonal) among five cattle breeds.

Breed	Brahman	Braunvieh	Gyr	AFB	Simmental
Brahman		0.46	0.14	0.38	0.43
Braunvieh	0.37		0.44	0.09	0.13
Gyr	0.13	0.36		0.36	0.42
AFB	0.31	0.08	0.30		0.09
Simmental	0.35	0.12	0.34	0.09	

**FIGURE 2:**
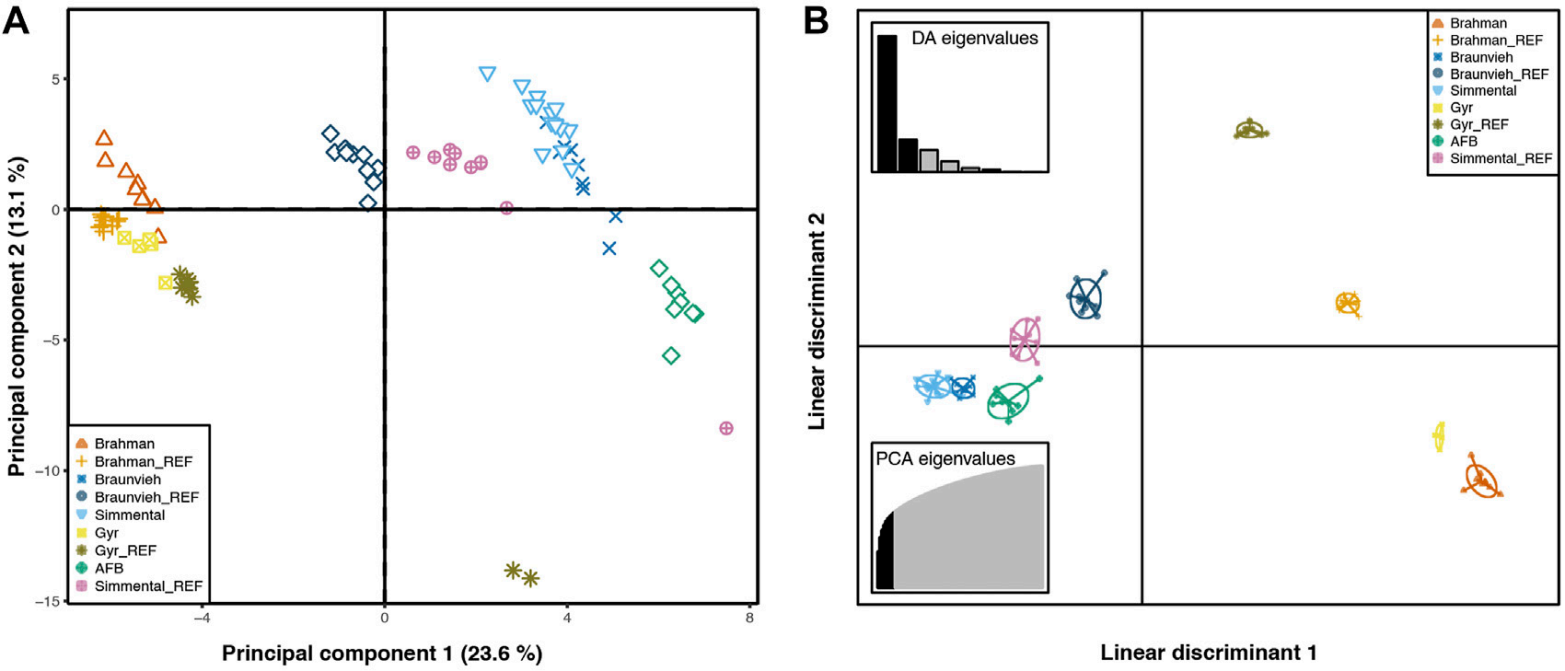
Principal component analysis (PCA) and Discriminant analysis of principal components (DAPC) plots. Samples belong to a reproductive cattle herd that comprises with Brahman (*N* = 9), Braunvieh (*N* = 9), Gyr (*N* = 5), and Simmental (*N* = 15) breeds; a locally adapted creole cattle, the Arequipa Fighting Bull (AFB, *N* = 9); and 40 genotype samples from reference breeds included in the analyses (subscript with _REF). Symbols and colors indicate breed affiliation, each symbol represents an individual. **(A)**. For PCA plot, the x- and y-axes are indicated by the first and second components, respectively, and the values in parentheses show the percentages of total variance explained. **(B)**. For DAPC plot, the scatterplot shows only the first two linear discriminants of the analysis.

Figure 2 presents the result of PCA and DAPC analysis performed to visualize individual relationships among populations. Individuals were grouped according to their breed origins. The first and second component accounted for a total of 23.60% and 13.10%, respectively. PCA and DAPC showed a low differentiation among the AFB, Braunvieh, and Simmental populations, while the Brahman and Gyr herds are clearly separated from the other three populations. In the PCA a substructure was observed corresponding to samples from Gyr, from the reference populations. The populations included in this study come from different selection environments. Brahman and Gyr individuals have been selected for tropical climates. The Simmental and Braunvieh groups have been selected in template environments, while AFB individuals have been mainly selected under artificial selection pressure.

**FIGURE 3:**
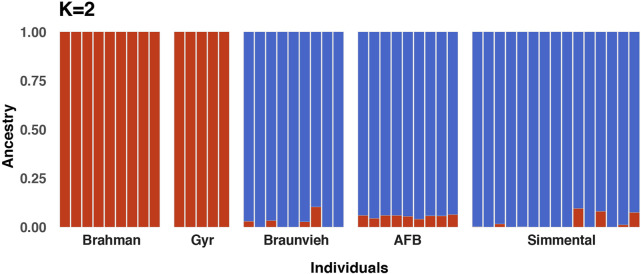
Population structure using 16,345 SNPs for five cattle breeds consisting of 47 individuals. Admixture analysis showing the proportions of ancestral populations for *K* = 2, each vertical bar exemplifies an individual.

A graphic representation of cluster structure analysis is depicted in Figure 3. Based on the ΔK value, *K* = 2 was the most optimal number for the inferred genetic structure of the populations (Supplementary Figure S1). At *K* = 2 a considerable source of variation among cattle breeds was perceptible. Cluster 1 comprised of the Brahman and Gyr breed groups (*N* = 14 genotypes), whereas cluster 2 consisted of the Braunvieh Simmental, and AFB cattle groups (*N* = 33 genotypes). The Brahman and Gyr populations displayed a separated cluster, whereas the Braunvieh, and AFB, and Simmental populations presented similar genetic construction.

**FIGURE 4:**
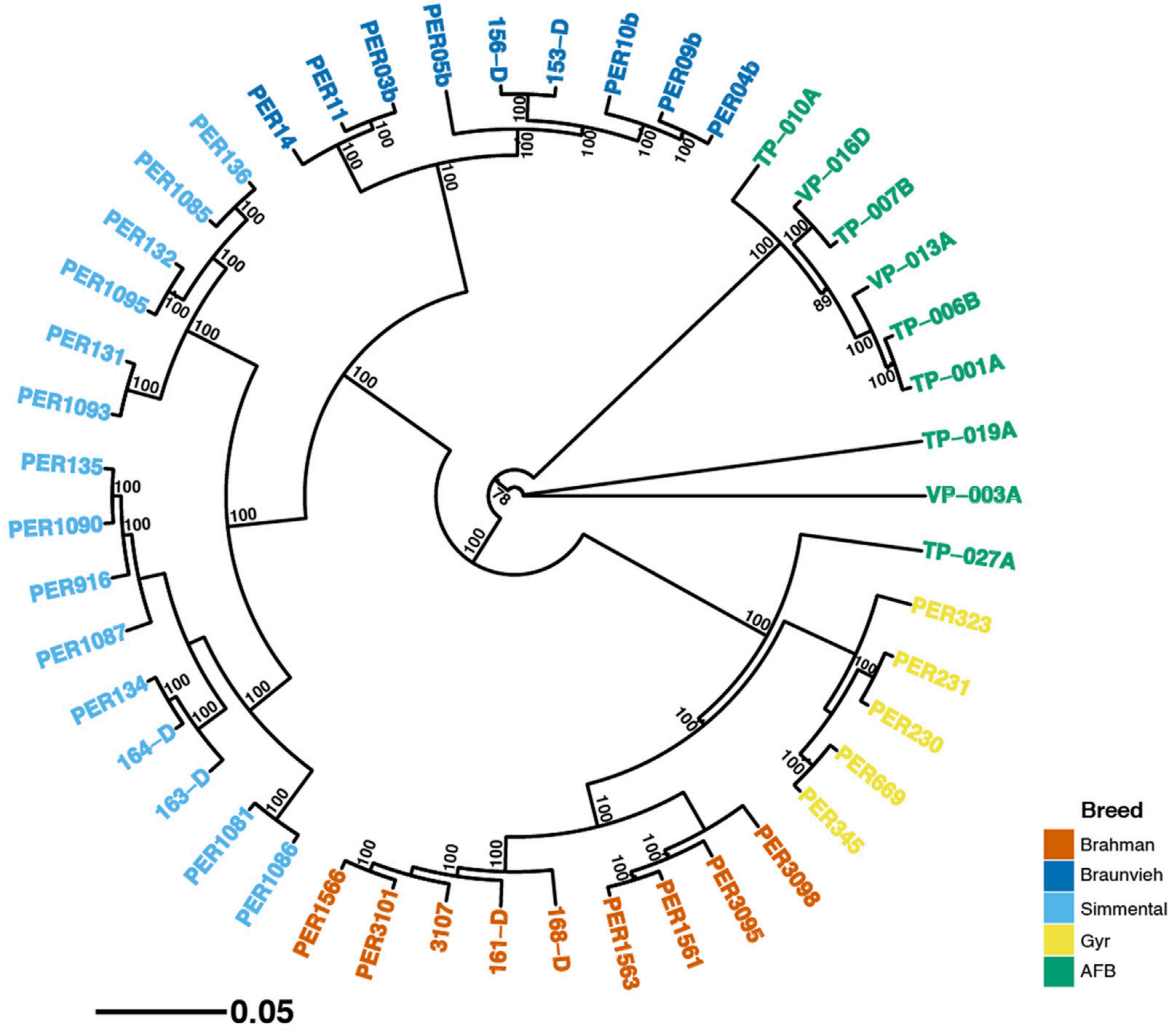
Phylogenetic relationship constructed using a neighbor-joining tree from a dataset of 80,178 SNPs in five breeds. Numbers above the branches represent bootstrap values, with only values higher than 70% shown.

A neighbor-joining tree was constructed from SNPs (Figure 4), displaying bootstrap support greater than 70%. The first group is composed of fifteen Simmental individuals with 100% bootstrap support. The second group is composed of nine Braunvieh individuals with 100% bootstrap support. Nine individuals AFB composed the third group. In concordance with the principal coordinate analysis, these groups are together. The fourth group is composed of nine Braunvieh individuals with 100% bootstrap support. Five individuals Gyr composed the fifth group. These groups are together with 100% bootstrap support, also in agreement with principal coordinate analysis. However, an individual AFB (TP−027A) was integrated into this group of Brahman and Gyr with 100% bootstrap support.

## 4 Discussion

Investigating genetic diversity parameters of populations is critical for developing future breeding objectives (Notter, 1999). Here for the first time, we examined population genetic structure of a group of animals that are been used as a genetic nucleus in Peru, as well as a group of PCC (AFB). Our results of Pn, MAF, He, Ho showed that the populations have a moderate genomic diversity. In this study, all the average values of MAF recorded in taurine cattle (i.e., Braunvieh, Simmental, and AFB) were higher than those recorded in zebu cattle (i.e., Brahman and Gyr). This result might be due to the low representation of zebuine cattle breeds in the SNP genotyping array used (Chagunda et al., 2018). Most of the bovine SNP panels available have been developed of the sequences of individuals belonging to European bovine breeds (European Cattle Genetic Diversity Consortium et al., 2006; The Bovine Hapmap Consortium et al., 2009). This might explain why the observed polymorphism of our SNP data set was higher in the Braunvieh, Simmental, and AFB breeds.

Previous studies have shown that breeding practices have a great effect on reducing genetic diversity, leading to a lower level of genetic diversity in selected germplasm compared with wild varieties (Tisdell, 2003; Zenger et al., 2007). Interestingly, our genetic diversity analysis with the four specialized breeds from the nucleus herd seems to agree. We observed a significantly higher genetic diversity level in the AFB group breed than in the specialized breeds, which is similar from those reported in previous studies (Giovambattista et al., 2001; Egito et al., 2007; Edea et al., 2015). In the case of AFB, it showed the highest levels of He and one of the highest for Ho. For these cattle population, there is a marked effect due to mating control by breeders, which can certainly play an important role (Hidalgo et al., 2015; Delgado et al., 2019). Creole breeds are primarily used in the Peruvian livestock systems to establish crosses with other species of *B. taurus*, particularly Brown Swiss and Simmental in high Andean areas (Primo, 1992; Quispe, 2016). Considering AFB, the mean value of Ho (0.42) obtained in this study is lower than that (0.77) reported by Martínez et al. (2015) in Costa Rica, (0.75) Lirón et al. (2006) in Argentine and Bolivian Creole Breeds, (0.68) Egito et al. (2007), in Brazil, (0.67) Ginja et al. (2010) in Portuguese Native Cattle, (0.70) Acosta et al. (2012) in Cuban cattle breeds. However, most of these studies are also in creole cattle from Latin America where the values greatly differ from ours. One explanation for these differences is that our study was based on SNP markers, whereas the other studies used microsatellite markers. As population genetic statistics can easily be applied to SNPs because they are often bi-allelic, however, a greater number of polymorphic loci may be required to match the power of multi-allelic SSR loci (Guichoux et al., 2011; Laoun et al., 2020). Also, the reduced Ho of the AFB may be explained on the fact that these individuals, compared to other local breeds, go through a process of strict artificial selection as growers always look for fighting traits. AFB are always part of the traditional bullfight activity of Arequipa. It should be noted that the Brahman and Gyr breed presented the lowest levels of He and Ho. This lower level of heterozygotes is generally interpreted as a deviation from random mating (Zeng et al., 2013; Lamkey and Edwards, 2015).

Regarding the content of Pn, a study in six breeds including Simmental, determined an average proportion of polymorphic SNPs of 79% (Dadi et al., 2012), while in this study was 79.72%. F_IS_ presented an average value of −0.04, which ranged from 0.03 (AFB) to −0.07 (Brahman and Braunvieh). So, this negative F_IS_ values could indicate that the population was in outbreeding (Caballero and Toro, 2002). In addition, mating could be occurring between individuals from different populations (Wright, 1965; Chesser, 1991). The F_IS_ value was negative for the studied breeds of the reproductive herd, where Brahman and Braunvieh had the lowest F_IS_ values, suggesting an excess of heterozygotes and a lack of population structure (Tantia et al., 2006). This could be due to the small sample population size.

According to the AMOVA results (Table 2), the proportion of genetic variability attributable to the difference variation among populations, and within individuals was 24.29% and 75.71%, respectively. These results implied lower genetic differentiation among breeds than within breeds maintained at EEA Donoso. Similar studies have reported lower values for variation across populations (Cañón et al., 2001; Lirón et al., 2006; Egito et al., 2007). Lirón et al. (2006) reported that 8.8% of the total genetic variation corresponded to differences between populations (zebu and taurine breeds), while 91.2% was explained by differences between and within individuals. Cañón et al. (2001) indicated that about 7% of the total genetic variation corresponded to differences between racial groups, while the remaining 93% corresponded to differences between and within individuals. On the other hand, Egito et al. (2007) reported a value of 12% for genetic variation attributable to differences between breed groups. The higher value obtained in the present study may be linked to the characteristics of the sampling (Kitada et al., 2021). The AFB breed group is made up of highly heterogeneous animals, which magnifies the within-group (within individual) variance compared to the between-group (among population) variance. Many of these groups are also highly related to each other (i.e., Gyr-Brahman pair, Simmental-Braunvieh pair), which is further confirmed in the population structure analyses. The little degree of variation is consistent with the F_ST_ for AFB-Braunvieh pair (0.08) and AFB-Simmental pair (0.09).

Table 3 showed that the lowest genetic distance (0.08) was observed for the AFB and Braunvieh. Similarly, for the AFB and Simmental breeds the genetic distance was low (0.09). These values close to zero indicate that these breeds shared their genetic material through breeding. Likewise, Figure 2 showed that Simmental, Braunvieh, and AFB grouped closely to each other. In addition, many individuals of AFB possess traits of Braunvieh and Brown Swiss as they are also employed for beef and milk production. Also, concordant to the genetic distance, PCA and DAPC indicated that Brahman and Gyr are closely related. The share of genetic material between Brahman and Gyr can be explained as they belong to the same species, *B. indicus*. On the contrary, higher genetic distances were observed between Brahman with 1) Braunvieh (0.37), 2) Simmental (0.35), 3) and AFB (0.31), indicating some degree of isolation between these breeds, that is, they are not currently breeding with one another. This could be because the region where the AFB samples were collected is located in the southern parts of the country, where climatic conditions are cold. Hence, European breeds such as Brown Swiss, Braunvieh, Simmental, Overo negro, Jersey, etc., are more commonly used in these regions because of the cold climate, whereas Zebuine breeds, such as the Brahman and Gyr, are preferably used in the Amazon region of the country, in the north. Our phylogenetic reconstruction is in concordance with ADMIXTURE analysis and genetic distances. We identified that the AFB breed is closer to Braunvieh than the Simmental breed and others. Arbizu et al. (2022) examined the relationship between PCC and other *B. taurus* with an analysis of the mitochondrial genome and validated these relationships, probably influenced by the high introgression and crossing over. Also, a strong relationship between Gyr and Brahman breeds was identified.

We analyzed the genetic structure of the AFB cattle by using SNP markers. This information will be valuable to our farmers as well as future studies. The results of this study provide some insight that AFB can become a separate breed in the future. The analysis also provides evidence for two subgroups within the AFB group (Figure 4), with one level higher of genetic differentiation than the other one. Also, this new information of a Peruvian reproductive cattle herd would offer valuable information to establish a genetic nucleus herd and modern breeding programs. In addition, we expect molecular tools become widely employed in favor of the cattle industry in Peru.

## 5 Conclusion

We here determined for the first time the genetic diversity and population structure of a Peruvian cattle herd using SNP data. Braunvieh breed possessed the highest genetic diversity while Brahman the lowest. Most of the variance occurs within individuals among the five breeds evaluated in this study. A total of two clusters were identified, showing, as expected, a clear separation between *B. indicus* (Brahman and Gyr) and *B. taurus* breeds (Braunvieh, AFB and Simmental). Interestingly, the AFB was placed in a single cluster, providing evidence that this may be considered a breed as farmers from Arequipa breed their animals in favor of fighting traits. Additional work is needed to also characterize other cattle herd of INIA located in San Martin region. We hope this work will pave the way towards developing a modern cattle breeding program in Peru.

## Data Availability

The original contributions presented in the study are publicly available. This data can be found here: https://datadryad.org/stash/share/xHP03DhsK4QVn2wh8QZzaOCGEBUYN1Kg5Pnuj2xkqIo.
