# Supplementary material for: Genetic diversity and population structure of a Peruvian cattle herd using SNP data
Source: Front Genet. 2023 Mar 10;14:1073843. doi: 10.3389/fgene.2023.1073843 (PMC10036791; doi:10.3389/fgene.2023.1073843)

Supplementary Figure S1. Number of populations (K) inferred by 10-fold cross validation. K ranges from 1 to 10.

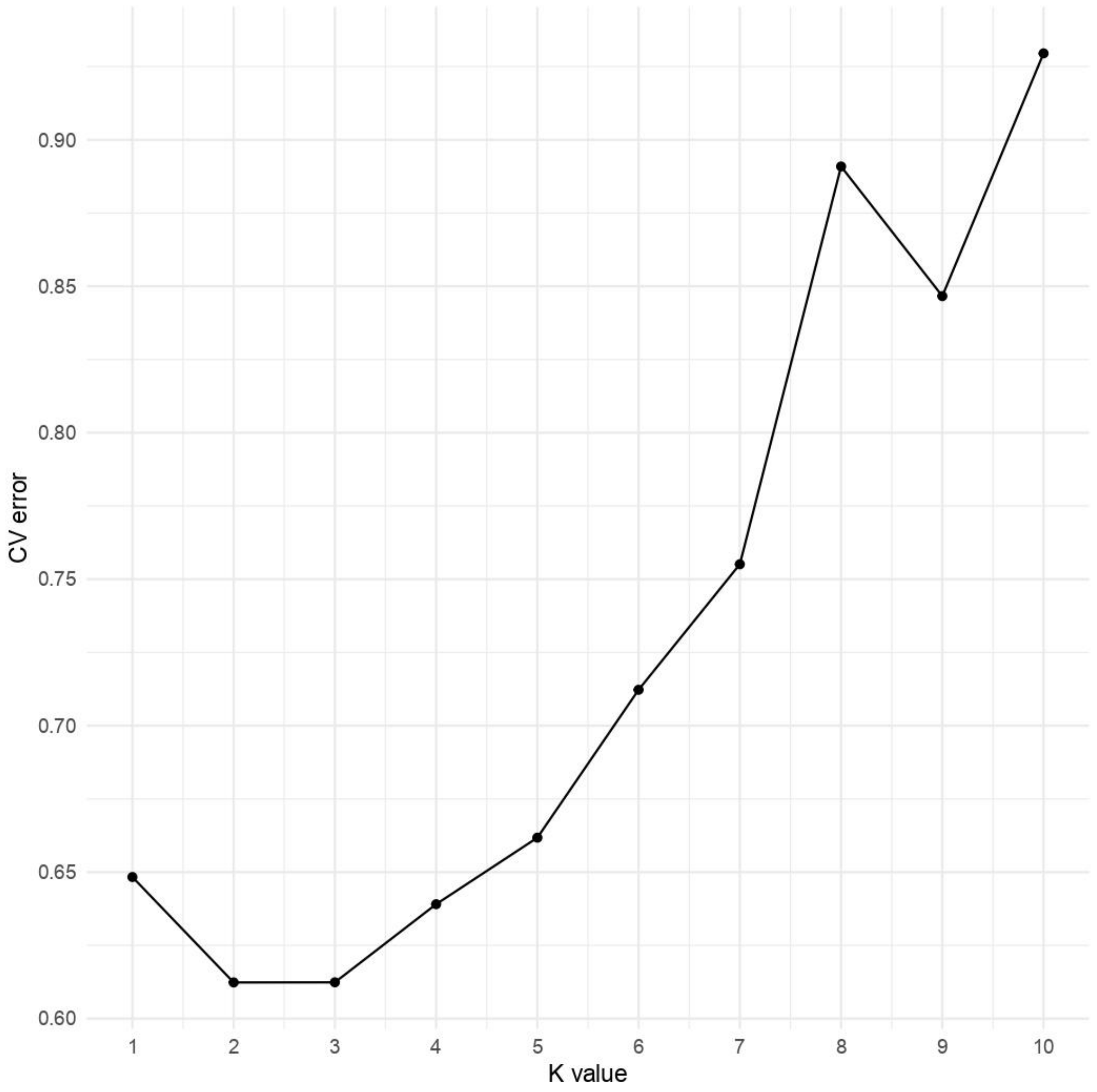

Supplement: Supplementary file 2 [file Image1.pdf]
